# Multi-Omics Analysis Detects Novel Prognostic Subgroups of Breast Cancer

**DOI:** 10.3389/fgene.2020.574661

**Published:** 2020-10-15

**Authors:** Quang-Huy Nguyen, Hung Nguyen, Tin Nguyen, Duc-Hau Le

**Affiliations:** ^1^Department of Computational Biomedicine, Vingroup Big Data Institute, Hanoi, Vietnam; ^2^Faculty of Pharmacy, Dainam University, Hanoi, Vietnam; ^3^Department of Computer Science and Engineering, University of Nevada, Reno, Reno, NV, United States; ^4^School of Computer Science and Engineering, Thuyloi University, Hanoi, Vietnam

**Keywords:** breast cancer, PAM50 subtypes, multi-omics, molecular subtypes, biomolecular markers

## Abstract

The unprecedented proliferation of recent large-scale and multi-omics databases of cancers has given us many new insights into genomic and epigenomic deregulation in cancer discovery in general. However, we wonder whether or not there exists a systematic connection between copy number aberrations (CNA) and methylation (MET)? If so, what is the role of this connection in breast cancer (BRCA) tumorigenesis and progression? At the same time, the PAM50 intrinsic subtypes of BRCA have gained the most attention from BRCA experts. However, this classification system manifests its weaknesses including low accuracy as well as a possible lack of association with biological phenotypes, and even further investigations on their clinical utility were still needed. In this study, we performed an integrative analysis of three-omics profiles, CNA, MET, and mRNA expression, in two BRCA patient cohorts (one for discovery and another for validation) – to elucidate those complicated relationships. To this purpose, we first established a set of CNAcor and METcor genes, which had CNA and MET levels significantly correlated (and anti-correlated) with their corresponding expression levels, respectively. Next, to revisit the current classification of BRCA, we performed single and integrated clustering analyses using our clustering method PINSPlus. We then discovered two biologically distinct subgroups that could be an improved and refined classification system for breast cancer patients, which can be validated by a third-party data. Further studies were then performed and realized each-subgroup-specific genes and different interactions between each of the two identified subgroups with the age factor. These findings can show promise as diagnostic and prognostic values in BRCA, and a potential alternative to the PAM50 intrinsic subtypes in the future.

## Introduction

The unprecedented proliferation of recent large-scale and multi-omics databases of cancers has given us many new insights into genomic and epigenomic deregulation in cancer discovery in general (Rappoport and Shamir, 2018). Accordingly, DNA copy number aberration (CNA) or mutations, resulting in genomic alteration, play vital roles in cancer occurrence and progression (Kim et al., 2018); meanwhile, DNA methylation (MET), resulting in epigenetic regulation of the cancer genome, is thought to make considerable contributions to the heterogeneity of cancer (Yang et al., 2019). Especially, with a highly heterogeneous disease like breast cancer (BRCA), it is clearly no exception (Luen et al., 2016; Karsli-Ceppioglu et al., 2017). Specifically, CNA profiling using CGH and SNP microarrays in prior studies has revealed hot spots of CNA in cancer genomes (Russnes et al., 2010; Huang et al., 2013; Endesfelder et al., 2014), such as, the frequent copy number gains have involved chromosomes 1q, 6q, 8q, 11q, 16q, 17q, 19, and 20q, whereas common deletion of copy number at 6q, 16q, 17p, and 22q in BRCA (Richard et al., 2000). Several oncogenes and tumor suppressor genes such as *HER2* (also known as *ERBB2*), *c-Myc*, *CCND1*, and *TP53* have been altered by CNA and exerted their key regulatory functions in both progression and prognosis of BRCA (Richard et al., 2000). In addition, previous studies have found several mutated epigenetic genes, partaking in establishing and maintaining epigenetic patterns, such as *MLL3* or *MLL2* mutations in BRCA (Stephens et al., 2012), or a recurrent epigenetic inactivation of *BRCA1* by epigenetic mechanisms in sporadic BRCA (Dobrovic and Simpfendorfer, 1997; Rice et al., 2000).

However, we wonder whether or not there exists a systematic connection between CNA and MET? And if so, what is the role of this connection in BRCA tumorigenesis and progression? In addition, the PAM50 intrinsic subtypes of BRCA (Parker et al., 2009) [Luminal A (LumA), Luminal B (LumB), Basal-like, HER2 over-expressed (HER2), and Normal-like], which are developed based on a 50-gene mRNA expression profile, have gained the most attention from BRCA experts. However, this classification system manifests its weaknesses including low accuracy as well as a possible lack of association with biological phenotypes, and even further investigations on their clinical utility were still needed (Untch et al., 2015). Looking back to the past, there are many publications attempting to reclassify breast tumors based on other omics data types such as miRNA arrays (Blenkiron et al., 2007; Bhattacharyya et al., 2015), copy number variations (Andre et al., 2009), or integration of different omics datasets (Shen et al., 2009; Curtis et al., 2012). Each of them has proposed various classification systems that have various agreements with the traditional classification, but collectively have implication for the existence of finer patient subgroups than the classical PAM50 subtypes (Dawson et al., 2013). Also, previous similar works such as Xia Y. et al. (2019) or Shi et al. (2015), focusing only on correlation analysis between CNA and mRNA expression, or de Almeida et al. (2019), focusing only on correlation analysis between MET and mRNA expression, discovered molecular mechanisms, potential biomarkers hidden in BRCA. Yet, a correlation between CNA, MET and corresponding mRNA, and an integrative computational approach using the three profiles (CNA, MET, and mRNA) was not ascertained to stratify BRCA patients.

**FIGURE 1:**
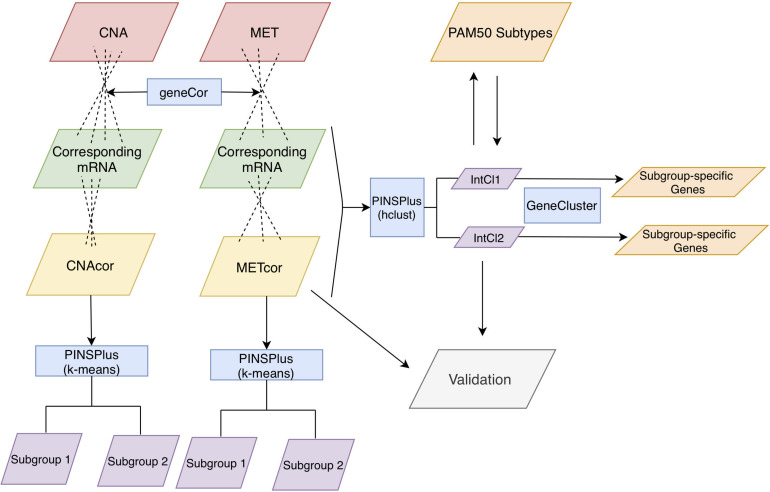
Analysis protocol. First, CNA, MET, and mRNA datasets were inputted to the function “*geneCor*” to identify a list of CNAcor and METcor genes. Then, we detected prognostic subgroups for individual CNAcor and METcor datasets, and a combination of CNAcor + METcor + mRNA through single and integrated analyses using PINPlus, respectively. Next, several analysis results (i.e., relationships between CNAcor or METcor with their corresponding mRNA, association of CNAcor or METcor with overall survival (OS) of BRCA patients, integrative clustering analysis, and comparison between the PAM50 subtypes and our integrative subgroups) were validated by an independent dataset. Finally, we examined the relationships between our classification system and the PAM50 subtypes before characterizing our subgroups using the R package GeneCluster. hclust, hierarchical clustering method.

In this study, we employed three-omics profiles, including CNA, MET, and mRNA expression levels in a cohort of BRCA patients, which were part of the TCGA project (Cancer Genome Atlas Research Network et al., 2013) and downloaded by cBioPortal (Cerami et al., 2012; Gao et al., 2013) to elucidate those complicated relationships. To this purpose, we first established a set of CNAcor and METcor genes, which were the CNA and MET ones significantly correlated with their corresponding expression levels, respectively, indicating the co-dysregulation of transcriptomics by CNA and MET aberrations. Next, to revisit the current classification of BRCA, we envisioned our classification system in the context of multi-omics, in which the first omics dataset was the mRNA expression, which was the only phenotype created the PAM50 intrinsic subtypes, combining with the later omics datasets including the profiles of CNAcor and METcor (Figure 1). As a result, single and integrated clustering analyses using our clustering tool PINSPlus (Nguyen et al., 2017, 2018) discovered two biologically distinct subgroups that could be as an improved and refined classification system for BRCA patients.

## Materials and Methods

### Materials

The two breast cancer cohorts [i.e., discovery (Cancer Genome Atlas Network, 2012) and validation (Cancer Genome Atlas Research Network et al., 2013)] used in our analysis are described in Table 1. These datasets were part of the TCGA project (Cancer Genome Atlas Research Network et al., 2013) and downloaded by cBioPortal (Cerami et al., 2012; Gao et al., 2013).

**TABLE 1:** Description of two breast cancer cohorts.

	Omics data	Platform	Description
Discovery data (Cancer Genome Atlas Network, 2012)	mRNA	Agilent microarray	A continuous matrix whose columns (the number of samples) are 526 samples and rows (the number of genes) are 5,961 genes.
	CNA	Affymetrix SNP6	A continuous matrix whose columns (the number of samples) are 778 samples and rows (the number of genes) are 20,871 genes.
	MET	Illumina Infinium HumanMethylation 27 platform	A continuous matrix whose columns (the number of samples) are 311 samples and rows (the number of genes) are 12,328 genes.
	Clinical data		Samples: 825 Overall survival (OS) status was defined as overall survival status (deceased or living), whereas OS time was defined as the time of diagnosis to the time of death or last follow up (unit: month). The follow-up time OS was truncated to 234 months.
Validation data (Cancer Genome Atlas Research Network et al., 2013)	mRNA	Agilent microarray	A continuous matrix whose columns (the number of samples) are 529 samples and rows (the number of genes) are 16,557 genes.
	CNA	Affymetrix SNP6	A continuous matrix whose columns (the number of samples) are 1,080 samples and rows (the number of genes) are 24,776 genes.
	MET	Illumina Infinium HumanMethylation 450 platform	A continuous matrix whose columns (the number of samples) are 778 samples and rows (the number of genes) are 16,474 genes.
	Clinical data		Samples: 1,108 The follow-up time OS was truncated to 283 months.

### Data Acquisition and Preprocessing

The preprocessing strategies for three profiles (i.e., mRNA, CNA, and MET) from the discovery data (Cancer Genome Atlas Network, 2012) were implemented as below. First, we removed patients whose gender was male or unknown because they were minor cases. Second, we matched the sample labels shared among the three profiles and clinical data and obtained 292 matched patients. Third, we filtered out genes with more than 50% missing values and then imputed the remaining missing values using the k-nearest neighbor algorithm (Batista and Monard, 2002) that is implemented in the function data.imputation function of the CancerSubtypes Bioconductor package (version 1.14.0) (Xu et al., 2017).

### Identification and Examination of the Relationship of CNAcor and METcor Genes

First, we calculated the Pearson’s correlation coefficient between MET and mRNA, as well as between CNA and mRNA using the matched data. This analysis helped to examine the global effects of genomics and/or epigenomic aberrations on transcriptomics changes. All significant correlation coefficients *r* (*P*-value ≤ 0.05) were then transformed to *Z* values using Fisher’s Z-transformation: *Z* = 0.5 ln[(1+*r*)/(1−*r*]). Second, we visualized the overall distribution of the resulting *Z* values that represent the relationship between MET-mRNA and CNA-mRNA. Third, the significance of the skewness for the Z distribution was further interrogated using the D’Agostino test (Timothy, 2017). The skewness overall indicates whether MET/CNA is correlated with mRNA. Considering those investigations conducted on examining the relationships between MET or CNA and corresponding mRNA data have been crucial in cancer researches, we now integratively developed the R package “*geneCor*”^1^ to perform the three above-mentioned tasks at once. Finally, due to a large number of genes in each of the two profiles, we only selected genes significantly associated with a prognostic value [i.e., OS of patients; *P*-value ≤ 0.05, logrank test (Bland and Altman, 2004)] using the function “*FSbyCOX*” in the package CancerSubtypes (version 1.14.0) (Xu et al., 2017).

### Expression of CNAcor and METcor Genes With OS in BRCA

We independently related the expression of each CNAcor gene and each METcor gene to the OS of patients as described in a previous publication (Jin et al., 2019). To this end, for CNAcor or METcor genes, the median expression of each one was computed across the patients, then we received two groups of the patients: patients having the expression of genes was greater than the median value assigned to the first group “up-regulation”; meanwhile, the second group “down-regulation” dedicated to patients having the expression of genes was less than the median values. Next, we performed univariate Cox regression analysis (Andersen and Gill, 1982) to observe the association between the expression levels of individual CNAcor or METcor genes and survival rates of the patients. Finally, hazard ratios (HR) with 95% confidence intervals (95% CI), *P*-values [logrank test (Bland and Altman, 2004)] and *Q*-values [Benjamini-Hochberg procedure (Benjamini and Hochberg, 1995)] were reported. Genes were defined as significantly associated with OS if *P*-value ≤ 0.05 and *Q*-value ≤ 0.05.

### Single and Integrated Analyses

In previous works (Nguyen et al., 2017, 2018), we introduced a clustering method called PINSPlus (version 2.0.3), which is a fast, powerful and state-of-the-art tool, confirmedly outperformed many other advanced approaches in either single or integrated analysis of multi-omics profiles. For the setting of both single and integrated clustering analyses, the number of clusters *k* was set to be between 2 and 10. Otherwise, for compatibility with the selected clustering method in the PAM50 subtypes: hierarchical clustering method (Lance and Williams, 1967), we chose this clustering method for the integrative clustering task [“k-means” (Forgy, 1965) is default clustering method]. The remaining parameters were left at default for reproducibility as well as consistency with a selected clustering method (i.e., k-means) in a single analysis in Netanely et al. (2016) paper that is convenient to compare the results.

### Identification of Subgroup-Specific Genes and Enrichment Analysis

A previous study defined subtype-specific genes are the ones mutated mainly in the samples assigned to one single subtype than in the other subtypes (Cyll et al., 2017). Subsequently, those genes are features that reflect the difference between subgroups of heterogeneous cancers (Alizadeh et al., 2015; Cyll et al., 2017). To computationally detect subtype-specific genes, we built the R package GeneCluster^2^ that consulted the idea of the reference paper (Shen et al., 2012). In brief, given a gene from a list of genes of interest, it will be specifically distributed to either of the identified subgroups based on the mean values (e.g., CNA changes, MET changes, and expression levels). Then, a gene was considered as a subtype-specific one if *P*-value ≤ 0.05 using the one-way ANOVA test (Cancer Genome Atlas Research Network et al., 2013).

Next, we performed the enrichment analysis on the set of identified subtype-specific gene sets to assess the clinical relevance of the sample subgroups using the DAVID tool (da Huang et al., 2009a,b) (version 6.8)^3^. The significance of the terms and pathways was computed by the Fisher’s exact *P*-value (Cancer Genome Atlas Research Network et al., 2013) (the smaller the *P*-value, the more significant).

### Validation of the Discovery Results

We validated several analysis results using a third-party dataset of BRCA from TCGA (Cancer Genome Atlas Research Network et al., 2013). The preprocessing process is the same as above described for the discovery data apart from the removal of 30 pairs of genes, in which each pair shared the same name with each other in mRNA expression data owing to the lack of essential information to retain them. Besides, we also matched the sample labels among the three profiles and clinical data, and obtained 202 matched patients at the end of this process.

In addition, we also validated the above results of association of CNAcor or METcor with OS (i.e., sub-section “Expression of CNAcor and METcor Genes With OS in BRCA”) by using the KMplot website^4^ (Györffy et al., 2010).

## Results

### Identification and Examination of the Distribution of CNAcor and METcor Genes

**FIGURE 2:**
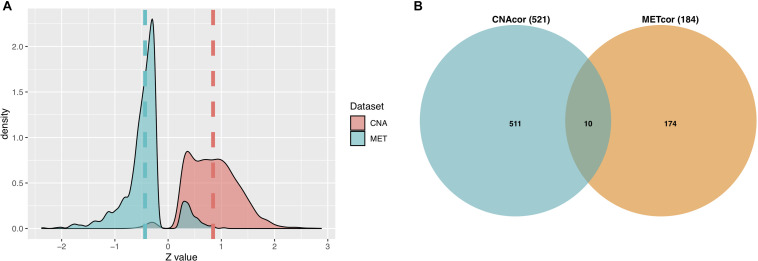
Characteristics of CNAcor and METcor genes in BRCA. **(A)** The distributions of the Z-scores that represent the correlation between MET or CNA with corresponding mRNA. **(B)** Intersection between the number of CNAcor genes and the number of METcor genes. CNA, DNA copy-number aberration and MET, epigenetic DNA methylation.

A total of 3,772 CNAcor genes and 2,118 METcor genes were identified by the R package “*geneCor*” (See Supplementary Table S1). As shown in Figure 2A, the distribution of CNAcor genes is significantly skewed to the right (skewness = 0.295, *P*-value = 1.879 × 10^–13^, D’Agostino test), suggesting that CNA genes are significantly correlated with gene expression. In contrast, the distribution of METcor genes are significantly skewed to the left (skewness = −0.211, *P*-value = 4.312 × 10^–5^, D’Agostino test), suggesting that MET genes are significantly anti-correlated with gene expression. Next, after performing the association of genes in each of two above sets with OS of patients to reduce the large quantity of CNAcor and METcor genes, a total of 521 CNAcor genes and 184 METcor genes (*P*-value ≤ 0.05 using logrank test) were preserved for downstream analyses. Besides, we found that there was a weak dependency between CNAcor and METcor since only 10 genes were intersected between these two sets (Figure 2B).

### CNAcor and METcor Genes With OS in BRCA

Univariate survival analysis was performed to assess the association between the expression levels of each gene from each of the two sets with OS of patients; then, we obtained 47 CNAcor genes and 13 METcor genes related to prognostic value in BRCA (*P*-value ≤ 0.05 and *Q*-value ≤ 0.05). Specifically, 28 CNAcor genes and four METcor genes with higher expression levels, and 19 CNAcor genes and nine METcor genes with lower expression levels had a significant association with poor outcomes. More details can be found in Supplementary Table S2. Next, after validating these results by the KMplot website, we observed five of 47 CNAcor genes including *CCNT1, MGAT5B, GNA13, KPNA2*, and *BSDC1*, and two of 13 METcor genes including *CAT* and *SLC25A5* were computationally validated prognostic markers (Table 2 and Supplementary Figure S1). This analysis indicated a relationship between some CNAcor and METcor genes with the pathophysiology of BRCA, and it could be of importance to predict the survival rates or severity of patients with BRCA.

**TABLE 2:** Computationally validated associations of CNAcor or METcor with OS of BRCA patients.

	Gene	HR (95% CI)	*P*-value	*Q*-value
CNAcor	CCNT1	3.266 (1.539–6.930)	0.001	0.018
	MGAT5B	0.336 (0.162–0.700)	0.002	0.021
	GNA13	3.195 (1.427–7.157)	0.003	0.018
	KPNA2	2.428 (1.192–4.945)	0.014	0.031
	BSDC1	0.441 (0.219–0.889)	0.021	0.042
METcor	CAT	0.451 (0.222–0.913)	0.025	0.036
	SLC25A5	2.165 (1.073–4.368)	0.031	0.036

*HR, Hazard ratio; 95% CI, 95% confidence interval.*

### Single and Integrated – Multi-Omics Data Analyses

**FIGURE 3:**
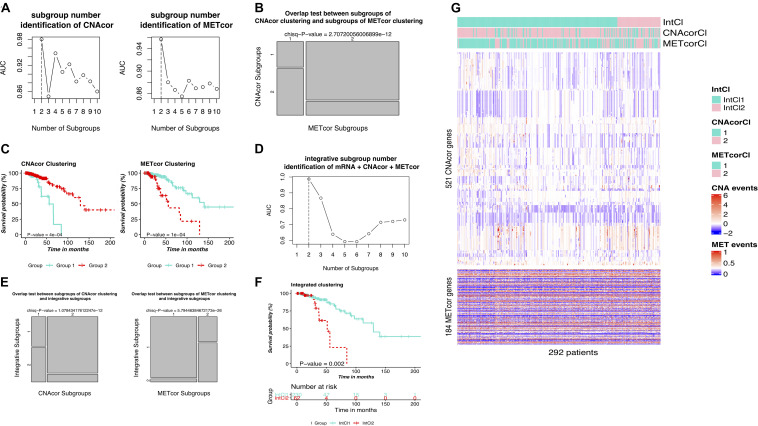
Identification of BRCA molecular subgroups using individual CNAcor and METcor genes for single clustering and CNAcor, METcor, and mRNA for integrative clustering. **(A,D)** AUC values obtained for each value of *k*. The optimal *k* is the value has the highest AUC value. **(A**-left, A-right, **D)** are the results of CNAcor clustering, METcor clustering, and integrated clustering, respectively. **(B)** Overlap test between subgroups of CNAcor and METcor clustering. **(E)** Overlap test between IntCl subgroups and CNAcor (left), METcor (right). **(C,F)** Kaplan-Meier survival curves for CNAcor subgroups **(C**-left), METcor subgroups **(C**-right), and **(F)** integrated subgroups. **(G)** Heatmaps that show the expression patterns of CNAcor subgroups (mid) and METcor subgroups (bottom) from integrated analysis by PINSPlus. The colored bars indicate the patient subgroups identified by single clustering based on CNAcor (CNAcorCl; mid), METcor (METcorCl; bottom), and integrated clustering (IntCl; top).

PINSPlus was employed to do the clustering task for CNAcor and METcor gene sets with cluster number *k* set to be between 2 and 10. All the settings of single and integrated analyses were processed as described in the section “Materials and Methods.” To this end, for single clustering results, determined *k* for each profile: CNA, *k* = 2 (AUC = 0.980; Figure 3A/left); MET, *k* = 2 (AUC = 0.957; Figure 3A/right). Interestingly, the optimal number of patient subgroups for each profile was both two, and they were significantly overlapped with each other (*P*-value = 2.707 × 10^–12^; χ2 test; Figure 3B), more strengthening our belief on the existence of the related regulation of CNAcor and METcor in BRCA. We further implemented survival analysis for each identified subgroup by CNAcor and METcor, and revealed that there were statistically significant differences in OS in the two subgroups of CNAcor dataset (*P*-value = 4 × 10^–4^; Figure 3C/left) and METcor dataset (*P*-value = 1 × 10^–4^; Figure 3C/right).

Next, we used PINSPlus to perform the integrated clustering analysis for the genomic data regarding CNAcor, METcor and mRNA gene sets with cluster number *k* from 2 to 10. As a result, the best value *k* = 2 (AUC = 0.987; Figure 3D), two subgroups IntCl1 (*n* = 230) and IntCl2 (*n* = 62), was detected, consistent with the single clustering results for individual CNA and MET datasets, respectively (*P*-value = 1.078 × 10^–12^ and 5.794 × 10^–26^, respectively; χ2 test; Figure 3E). In addition, the survival analysis revealed significantly different prognostic outcomes between the two subgroups, in which the patients in the IntCl2 had the worse survival rates than those in the IntCl1 (HR = 4.248; 95% CI = 1.833–9.847; *P*-value = 0.002; logrank test; Figure 3F). Obviously, the *P*-values ≤ 0.05 shown in Figures 3C,F indicated that both single and integrative classification strategies using PINSPlus successfully found two distinct prognostic subgroups significantly correlated with BRCA patient outcomes. The single and integrated clustering results were visualized as heatmaps in Figure 3G. Also, cohort descriptions comprising age, tumor stage, metastasis status, ER status, PR status, number of positive lymph nodes, intrinsic PAM50 subtypes, and HER2 status for the BRCA patients reviewed between the IntCl1 and IntCl2 were provided in Supplementary Table S3.

### Validation of the Analysis Results

**FIGURE 4:**
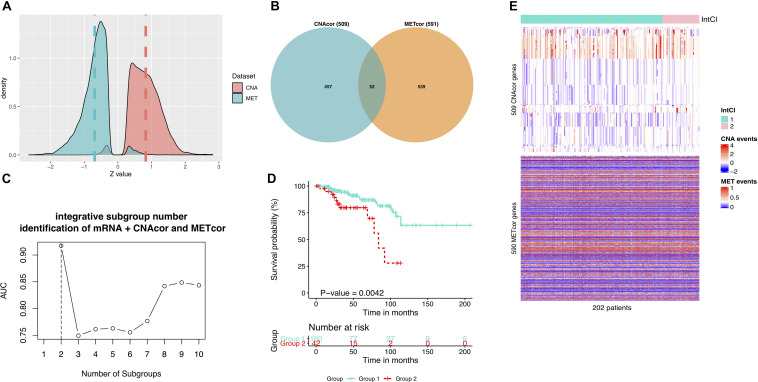
Validation results using a third-party BRCA data. **(A)** Z-score distributions of correlation between MET or CNA with corresponding mRNA. **(B)** Intersection between CNAcor genes and METcor genes. **(C)** The corresponding AUC values for each value *k*. The highest AUC value is for the value *k* = 2. **(D)** Kaplan-Meier survival curves for integrated subgroups. **(E)** Heatmaps that shows the expression patterns of CNAcor subgroups (mid) and METcor subgroups (bottom) from integrated analysis by PINSPlus.

To ensure that our findings were robust and consistent, we applied the same strategy to a third-party BRCA dataset. Consistent with the earlier analysis results, in the validation data, a total of 10,379 CNAcor genes and 9,471 METcor genes were identified by the R package “*geneCor.*” We also realized that the Z-score’s distribution of correlation coefficients between CNA and the corresponding mRNA was significantly skewed to the right (skewness = 0.225, *P*-value = < 2.2 × 10^–16^, D’Agostino test), whereas between MET and the corresponding mRNA was significantly skewed to the left (skewness = −0.260, *P*-value = < 2.2 × 10^–16^, D’Agostino test) (Figure 4A). Subsequently, we only retained 509 CNAcor and 590 METcor gene sets significantly associated with prognostic value using the same analysis protocol, and only 52 overlaps were recorded in these two sets (Figure 4B).

For clustering concern, we also determined that the two number of integrated subgroups was the best (AUC = 0.917; Figure 4C), in which the subgroup 2 had significantly poorer outcomes than the subgroup 1 in OS (HR = 3.279; 95% CI = 1.532–7.019; *P*-value = 0.004; logrank test; Figure 4D). Besides, the integrated clustering result were plotted as the heatmap in Figure 4E. This validation process proved that the strategy employed in the study is most likely to be efficient in prognostic subgroup pinpointing with various genomic and epigenomic regulation on the basis of CNAcor and METcor. In other words, the profile of CNAcor and METcor gene sets may help identify prognostic molecular subgroups on independent patient cohorts and data platforms.

### Molecular Characteristics of Integrated Subgroups

#### Comparing Resulting Subgroups to PAM50 Labels

**FIGURE 5:**
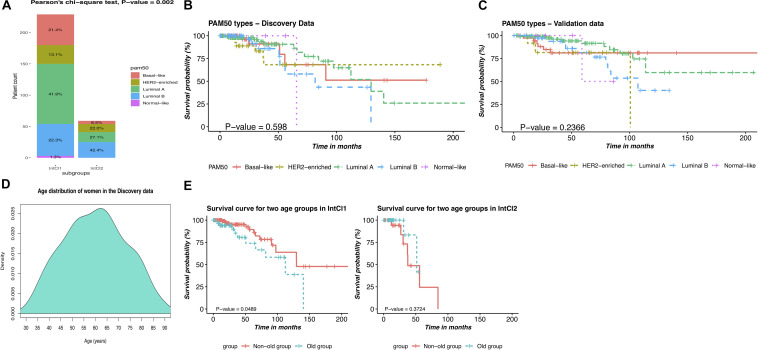
Comparison of the PAM50 subtypes and the integrative subtypes, and description of age-related risks of BRCA patients in each integrative subtype. **(A)** Distribution of the PAM50 intrinsic subtypes between the two IntCl. **(B)** Kaplan-Mayer survival curve for the PAM50 labels in the discovery data. **(C)** Kaplan-Mayer survival curve for the PAM50 labels in the validation data. **(D)** Age distribution of women in the discovery data. **(E)** Kaplan-Meier survival curves of patients in two age groups in the identified subgroups.

Next, we hypothesized that our two subgroups, the IntCl1 and IntCl2, had a closed relationship with the PAM50 label classes, and attempted to link the resulting partitions to these five intrinsic subtypes. Overall, the expression phenotype of the tumors in the IntCl1 was predominantly LumA (41.9%), followed by LumB (22.3%), basal-like (21.4%), HER2 (13.1%), and all of the normal class (1.3%), whereas the expression phenotype of the IntCl2 composed of LumB, LumA, HER2, and Basal-like, with the occurrence rates of 42.4%, 27.1%, 22.0%, and 8.5%, respectively (Figure 5A). These results suggest that the traditional classification does not capture well the variability among the BRCA samples, implying the finer subgroups could be undiscovered, possibly having clinical meaning.

#### The Integrative Clustering Predicts OS of BRCA Patients Better Than PAM50

We next sought to further interrogate the variability among the patients with BRCA by performing the survival analysis on the PAM50 classes. Surprisingly, comparison of Figures 3F, 5B for the discovery data and of Figures 4D, 5C for the validation data showed that the integrative clustering outperformed the PAM50 scheme in term of survival rates of the patients with BRCA, in which the patients in the IntCl1 had a significantly better survival probability than their counterparts in the IntCl2, with 85.05% survival at 5 years. According to this result, the weakness of Parker et al. (2009) classification system is revealed clearly from an integrated clustering analysis perspective, possibly due to the creation based on one single mRNA expression data.

#### Identification of Subgroup-Specific Genes and Enrichment Analysis Using DAVID Tool

To that end, we determined three lists of subgroup-specific genes in either of the integrative clustering, which was built based on mean mRNA expression levels (IntCl1: 1659 genes, intCl2: 1329 genes; Supplementary Table S4), mean methylation events (IntCl1: 53 genes, IntCl2: 51 genes; Supplementary Table S5), mean copy number changes (IntCl1: 149 genes, intCl2: 99 genes; Supplementary Table S6), and the results of the enrichment analysis using the DAVID tool were shown in Supplementary Tables S7, S8. From that, the major molecular properties for each of the integrative subgroups were revealed. The most notable feature of the IntCl1 was the overexpression of gene *MTF* and a substantial number of genes assigned to the CD gene family (*CD1c, CD22, CD58, CD82, CD93, CD96, CD160*, and *CD180*); frequent copy-number deletion of genes *MYOCD, RICH2, SAT2*, and *ZNF18;* hypermethylation of genes *SLC39A4, MAGEA10, UCP3, LGI4*, and *MYBPC3;* and was enriched most in mitochondrial membrane, structural constituent of ribosome by METcor genes (Supplementary Table S7), and intracellular part, cytoplasmic side of plasma membrane, heterocyclic compound binding by CNAcor genes (Supplementary Table S8). The IntCl2 was characterized by frequent copy-number amplification of genes *TUBD1, RPS6KB1, TMEM49*, and *PTRH2*; hypermethylation of genes *ALS2CR11, FAM89A, PTF1A*, and *GIPC2*; and was related most to development processes, single-multicellular organism process by METcor genes (Supplementary Table S7), and intracellular, catalytic activity by CNAcor genes (Supplementary Table S8).

Then, we strived to investigate further molecular events behind the two identified subgroups. In the IntCl1, BRCA samples had homogeneity of genes assigned to the CD gene family related closely to various immune processes (Janeway et al., 2001). A large number of overexpressed genes were highly associated with the B-cell receptor (*CD22*), or the T-cell receptor (*CD40* and *CD45/PTPRC*) and the upstream part of its signaling pathway (*LAT, LCP2, NCK1, FOS, MAP3K14, PAK4*, and *MAP3K8*) (Ogata et al., 1999). Interestingly, a simultaneous association of the elevated expression level of several genes with T-cell and natural killer-mediated cytotoxic activities was seen (*TNF, LCP2, NFATC1*, and *PIK3CD*) (Ogata et al., 1999). We also observed that several overexpressed immune-receptor-related genes went along with several overexpressed chemokine genes (*CXCL1, CXCL2, CXCL3*, and *CCL21*). The highest expressed gene among IntCl1-specific genes in Supplementary Table S4 was *MTF1* (ranked by mean expression value), whose mechanism included the regulation of the proper immune response (Grzywacz et al., 2015). For the IntCl2, many IntCl2-specific genes were enriched most in the development processes (Supplementary Table S7). Surprisingly, we continued to perform the same analyses above in the validation data and gained consistent results. More specifically, a list of 3993 IntCl1-specific EXP genes was shown in Supplementary Table S9, including a large number of genes belonging to the CD gene family. In contrast, IntCl2-specific genes were still closely related to development processes (Supplementary Table S10). Also, in a previous study (Xia Y. et al., 2019), the authors built Elastic Net prediction models to identify gene signatures as well as predict their subtype specificities, and realized that CD8 T-cell signature (Iglesia et al., 2016) had a validated association with Basal-like subtype-specific genes, potentially implying immunotherapy may be applicable to BRCA Basal-like samples (Xia Y. et al., 2019). As shown in Figure 5A, the Basal-like subtype was mainly distributed to the IntCl1 (21.4%) and also related to the immune system. This consistent finding could be a potential interpretation for why IntCl1 has associated with the immune system as well as a useful recommendation for oncologists to optimally select appropriate therapies for the IntCl1-assigned patients.

Finally, we sought to compare our two lists of subgroup-specific genes, related to genomic alterations (Supplementary Tables S4, S6), with the most widely used DNA-based gene panel Foundation One, which has been having 313 genes (accessed on Aug 13, 2020) being selected as cancer-related and reported to play central roles in tumorigenesis. As a result, we observed 34 subgroup-specific expression genes (bold red text, Supplementary Table S4) and four subgroup-specific CNAcor genes (bold red text, Supplementary Table S6) appeared in the gene panel. These results could clinically strengthen our findings on the association between lists of subgroup-specific genes with tumorigenesis in BRCA.

#### Prognostic Factor Identification

We also investigated the distribution of the initial diagnosis ages and the survival time of the patients in each cancer subgroup. As shown in Table 3, women of 58 or older had been at higher risk of having BRCA. Besides, we can see that the average survival time was 17.8 and 31.8 months in the IntCl2 and IntCl1, respectively. This result indeed demonstrated that we could totally anticipate the OS of patients between these two integrative subgroups. On top of that, the patients in the IntCl1 group an average initial diagnosis age and survival time of 58.6 years and 31.8 months, respectively, whereas in the IntCl2 group, the average initial diagnosis age was 64.8 years (relatively 6 years older than that of the IntCl1 group) and the average survival time was only 17.8 months. This may show that different integrative subgroups possess immensely different age-related risks and survival rates. To more clearly observe these risks in each integrative subgroup, the patients assigned to each subgroup were divided into two groups at 65-years threshold (peak in age distribution in Figure 5D), which meant having two age groups: non-old (age ≤65) and old (age >65).

**TABLE 3:** Average diagnosis ages and survival time of the BRCA patients in the integrative subgroups.

Subgroups	IntCl1	IntCl2
Average diagnosis age (year)	58.604	64.774
Average survival time (month)	31.772	17.826

We performed survival analyses on these age groups in each subgroup. As pictured in Figure 5E, the two age groups had a significant survival difference in the IntCl1 (*P*-value = 0.049; logrank test; Figure 5E/left); whereas, in the IntCl2, there was not a statistically significant difference in the survival rates between two age groups (*P*-value = 0.372; logrank test; Figure 5E/right). This indicated that the age factor might differently affect the ability to predict the survival probability in different molecular subgroups.

Regarding the results from Figure 5E, we realized that there existed different interactions between patients’ age and each of the two subgroups. Firstly, we observed the statistically significant difference between two age groups with regard to patient outcomes in the IntCl1, possibly owing to immune-related genes. Indeed, many previous studies were of interest to the interaction between the immune system and age factor in BRCA (Fuentes et al., 2017; Li et al., 2020; Xu et al., 2020). Especially, Li et al. (2020) indicated that there was a positive correlation of aging with i) the lower median immune/stromal scores and ii) the lower OS of BRCA patients. In contrast, an insignificant correlation of the IntCl2’s patient survival with age factor was monitored. Further performing correlation analysis of the IntCl2 with other clinical features in terms of survival rates, all of them were statistically insignificant again (Supplementary Figure S2). One potential explanation might be due to the small number of patients in this subgroup (i.e., 62 BRCA patients).

## Conclusion and Discussion

The unprecedented proliferation of recent large-scale and multi-omics databases of cancers provides numerous new insights into genomic and epigenomic dysregulation in cancer discovery (Rappoport and Shamir, 2018). Publicly available databases like TCGA, METABRIC, or GEO, which are common in the cancer research community, help better understand tumor heterogeneity, detect biomarker genes, and define hidden molecular mechanisms in multi-omics research (Xia Q. et al., 2019). Moreover, lines of previous evidence indicate notable relationships between CNA and mRNA, such as there is a high association of CNA with the development and progression of cancers by regulating gene expression level (Huang et al., 2017; Samulin Erdem et al., 2017; Zhou et al., 2017; Gut et al., 2018), as well as the similar regulatory associations between MET and mRNA (Herman and Baylin, 2003; Shen and Laird, 2013). Even a poor grasp of the additional biological complexity of breast tumors neglected at the expression level can be revealed at the DNA methylation level, possibly resulting in finer subgroups with clinical meaning (Rønneberg et al., 2011). With these concerns in mind, they motivate us to discover the relationships among the three pillars (mRNA, CNA, and MET) in BRCA, as well as compare our classification system to the PAM50 group.

In this study, we first used 292 BRCA patients in the TCGA database to establish CNAcor and METcor gene sets by computing the correlation of CNA and MET with their corresponding mRNA using the function “*geneCor.*” Subsequently, biomarker genes were detected, in which five CNAcor genes and two METcor genes were computationally validated prognostic markers following the recommendation of the KMplot dataset. By integrating three datasets mRNA, CNAcor, and METcor using the clustering tool PINPlus, we were able to efficiently and successfully stratify BRCA patients into two subgroups (IntCl1 and IntCl2) that reflected distinct molecular characteristics and their significant survival differences as well. Our findings were then tested on an independent dataset for validation.

For the molecular features of either of the integrative subgroups, a comparison of our integrative subgroups and the PAM50 scheme was implemented. Our analysis showed that discrimination among five PAM50 labels was unoptimistic. Fittingly, in agreement with (Netanely et al., 2016), we also found that two LumA and LumB label classes are distributed greatly into the two integrative subgroups. Moreover, also consistent with our result, the expression phenotype of LumA is the best prognosis subtype than the remaining PAM50 classes; however, when the author group of Dir Netanely (Netanely et al., 2016) reclusters these two subtypes, they reveal that LumA samples are divided into two separate subgroups whose outcomes of the BRCA patients are different significantly. Remarkably, one of the properties of the IntCl1 was a homogenous normal-like subgroup. The fact that the normal-like label is suspected as an artifact in the PAM50 subtypes, and this finding raises the possibility that we could exclude this artifact, although further studies are required. Furthermore, we proposed the tool “*GeneCluster*” in order to computationally explore subgroup-specific genes for each of the integrative subgroups. As a result, the IntCl1 exhibits distinct overexpression of immune-related genes, whereas the display of the IntCl2 is distinct hypermethylation of developmental genes.

Next, we further investigate molecular events behind the two subgroups as well as the distribution of the PAM50 subtypes within the two with regard to the CNA recurrent. As shown in Supplementary Figure S3, there is a positive relationship between the percentage of CNA burden category and the number of patients assigned to the IntCl2 (conversely, a negative relationship with the IntCl1). Interestingly, when linking these with the result in Figure 5A, a consistent finding is even reported between our work with previous work (Bland and Altman, 2004). Specifically, when an increase in the CNA burden category within the subgroups happens, it will lead to a rise in the incidence of the Luminal B label and a decrease in the rate of the Normal-like label. These results are a potential explanation of why a characteristic of the IntCl1 is the homogeneity of Normal-like subtype as well as a different distribution of the Luminal A and Luminal B label classes. Then, we try to fit the relationship between the identified subgroups with CNA and MET data across BRCA patients using a linear regression model. Consequently, we observed an insignificantly negative coefficient (i.e., coeff = −0.43, *P*-value = 0.07) for CNA and a significantly positive coefficient (i.e., coeff = 1.69, *P*-value = 7.65 × 10^–07^) for MET. In other words, this result indicates the relationship between the identified subgroups with CNA and MET data cannot be simply fitted by the linear regression model. That is the reason why our previous method PINSPlus (Nguyen et al., 2017, 2018) non-linearly integrates multiple -omics data for cancer subtyping. Furthermore, we compare the CNA burden between the two identified subgroups using *t*-Test (Two-Sample Assuming Unequal Variances). As a result, mean of CNA burden of the IntCl1 group (i.e., 0.11) is significantly lesser than that of the IntCl2 group (i.e., 0.16) (*P*-value < 0.01).

Last, our classification system differentiates amongst other papers from the selection of the clustering method in order to reclassify the BRCA patients. More specifically, we all know that the PAM50 scheme is initially advanced from Perou et al. (2000) classification with the hierarchical clustering method for mRNA expression data. Meanwhile, Mathews et al. (2019) used the topological data analysis or Netanely et al. (2016) used the k-means method, and so on, which help unveil new insights into BRCA patient re-classification; however, the papers use different clustering methods rather than using the hierarchical clustering method to subtype patients. When comparing the subtypes resulted from evaluated methods with subtypes from PAM50, it is rather difficult to determine if the markers or the method used help to improve the subtyping results. In this study, we used the same clustering method, i.e., hierarchical clustering, but in the background of a more advanced tool and under integrated analysis perspective. Clearly, our classification system is finer than the PAM50 groups with regard to survival probability estimation relying on integrated multi-omics implementation. In contrast, a more advanced tool like PINSPlus with the same clustering method can make sure that the identified subgroups have clinically meaningful features but are still consistent with clustering method used in the work of Parker et al.

In conclusion, multi-omics data integration of genomics, epigenomics, and transcriptomics helped us discover possible pathogenic mechanisms, as well as underscored a crucial role of DNA, CNA and MET in BRCA. In addition, using datasets consisting of these data types, we also detected two clinically relevant molecular subgroups with subgroup-specific features. These can pave the way for the development of accurate diagnostic tests and personalized treatments, and a potential alternative to the PAM50 intrinsic subtypes in the future.

## Data Availability Statement

The datasets presented in this study can be found in online repositories. The names of the repository/repositories and accession number(s) can be found in the article/Supplementary Material.

## Author Contributions

Q-HN conceived the idea and wrote the manuscript, which was edited by all co-authors, wrote the code, ran the model, and analyzed the output data. TN and HN coded the PINSPlus algorithm. D-HL supervised the work. All authors read and approved the final manuscript.

## Conflict of Interest

The authors declare that the research was conducted in the absence of any commercial or financial relationships that could be construed as a potential conflict of interest.

## Footnotes

1 https://github.com/huynguyen250896/geneCor

2 https://github.com/huynguyen250896/GeneCluster

3 https://david.ncifcrf.gov/home.jsp

4 http://kmplot.com/analysis/index.php?p=service&cancer=breast

## References

[B1] Alizadeh A. A. Aranda V. Bardelli A. Blanpain C. Bock C. Borowski C. (2015). Toward understanding and exploiting tumor heterogeneity. *Nat. Med.* 21 846–853. 10.1038/nm.3915 26248267 PMC4785013

[B2] Andersen P. Gill R. (1982). Cox’s regression model for counting processes: a large sample study. *Ann. Stat.* 10 1100–1120. 10.1214/aos/1176345976

[B3] Andre F. Job B. Dessen P. Tordai A. Michiels S. Liedtke C. (2009). Molecular characterization of breast cancer with high-resolution oligonucleotide comparative genomic hybridization array. *Clin. Cancer Res.* 15 441–451. 10.1158/1078-0432.ccr-08-1791 19147748

[B4] Batista G. Monard M.-C. (2002). *A Study of K-Nearest Neighbour as an Imputation Method*, Vol. 30 Amsterdam: IOS Press, 251–260.

[B5] Benjamini Y. Hochberg Y. (1995). Controlling the false discovery rate: a practical and powerful approach to multiple testing. *J. R. Stat. Soc. Ser. B* 57 289–300. 10.1111/j.2517-6161.1995.tb02031.x

[B6] Bhattacharyya M. Nath J. Bandyopadhyay S. (2015). MicroRNA signatures highlight new breast cancer subtypes. *Gene* 556 192–198. 10.1016/j.gene.2014.11.053 25485717

[B7] Bland J. M. Altman D. G. (2004). The logrank test. *BMJ* 328 1073–1073. 10.1136/bmj.328.7447.1073 15117797 PMC403858

[B8] Blenkiron C. Goldstein L. D. Thorne N. P. Spiteri I. Chin S. F. Dunning M. J. (2007). MicroRNA expression profiling of human breast cancer identifies new markers of tumor subtype. *Genome Biol.* 8:R214. 10.1186/gb-2007-8-10-r214 17922911 PMC2246288

[B9] Cancer Genome Atlas Network (2012). Comprehensive molecular portraits of human breast tumours. *Nature* 490 61–70. 10.1038/nature11412 23000897 PMC3465532

[B10] Cancer Genome Atlas Research Network Weinstein J. N. Collisson E. A. Mills G. B. Shaw K. R. Ozenberger B. A. (2013). The cancer genome atlas pan-cancer analysis project. *Nat. Genet.* 45 1113–1120. 10.1038/ng.2764 24071849 PMC3919969

[B11] Cerami E. Gao J. Dogrusoz U. Gross B. E. Sumer S. O. Aksoy B. A. (2012). The cBio cancer genomics portal: an open platform for exploring multidimensional cancer genomics data. *Cancer Discov.* 2:401. 10.1158/2159-8290.cd-12-0095 22588877 PMC3956037

[B12] Curtis C. Shah S. P. Chin S. F. Turashvili G. Rueda O. M. Dunning M. J. (2012). The genomic and transcriptomic architecture of 2,000 breast tumours reveals novel subgroups. *Nature* 486 346–352. 10.1038/nature10983 22522925 PMC3440846

[B13] Cyll K. Ersvær E. Vlatkovic L. Pradhan M. Kildal W. Avranden Kjær M. (2017). Tumour heterogeneity poses a significant challenge to cancer biomarker research. *Br. J. Cancer* 117 367–375. 10.1038/bjc.2017.171 28618431 PMC5537489

[B14] da Huang W. Sherman B. T. Lempicki R. A. (2009a). Bioinformatics enrichment tools: paths toward the comprehensive functional analysis of large gene lists. *Nucleic Acids Res.* 37 1–13. 10.1093/nar/gkn923 19033363 PMC2615629

[B15] da Huang W. Sherman B. T. Lempicki R. A. (2009b). Systematic and integrative analysis of large gene lists using DAVID bioinformatics resources. *Nat. Protoc.* 4 44–57. 10.1038/nprot.2008.211 19131956

[B16] Dawson S. J. Rueda O. M. Aparicio S. Caldas C. (2013). A new genome-driven integrated classification of breast cancer and its implications. *EMBO J.* 32 617–628. 10.1038/emboj.2013.19 23395906 PMC3590990

[B17] de Almeida B. P. Apolónio J. D. Binnie A. Castelo-Branco P. (2019). Roadmap of DNA methylation in breast cancer identifies novel prognostic biomarkers. *BMC Cancer* 19:219. 10.1186/s12885-019-5403-0 30866861 PMC6416975

[B18] Dobrovic A. Simpfendorfer D. (1997). Methylation of the BRCA1 gene in sporadic breast cancer. *Cancer Res.* 57:3347. 9269993

[B19] Endesfelder D. Burrell R. A. Kanu N. McGranahan N. Howell M. Parker P. J. (2014). Chromosomal instability selects gene copy-number variants encoding core regulators of proliferation in ER+ breast cancer. *Cancer Res.* 74 4853–4863. 10.1158/0008-5472.can-13-2664 24970479 PMC4167338

[B20] Forgy E. W. (1965). Cluster analysis of multivariate data : efficiency versus interpretability of classifications. *Biometrics* 21 768–769.

[B21] Fuentes E. Fuentes M. Alarcón M. Palomo I. (2017). Immune system dysfunction in the elderly. *An. Acad. Bras. Ciênc.* 89 285–299. 10.1590/0001-3765201720160487 28423084

[B22] Gao J. Aksoy B. A. Dogrusoz U. Dresdner G. Gross B. Sumer S. O. (2013). Integrative analysis of complex cancer genomics and clinical profiles using the cBioPortal. *Sci. Signal.* 6:l1. 10.1126/scisignal.2004088 23550210 PMC4160307

[B23] Grzywacz A. Gdula-Argasińska J. Muszyńska B. Tyszka-Czochara M. Librowski T. Opoka W. (2015). Metal responsive transcription factor 1 (MTF-1) regulates zinc dependent cellular processes at the molecular level. *Acta Biochim. Pol.* 62 491–498. 10.18388/abp.2015_1038 26336656

[B24] Gut A. Moch H. Choschzick M. (2018). SOX2 gene amplification and overexpression is linked to HPV-positive vulvar carcinomas. *Int. J. Gynecol. Pathol.* 37 68–73. 10.1097/pgp.0000000000000388 28700423

[B25] Györffy B. Lanczky A. Eklund A. C. Denkert C. Budczies J. Li Q. (2010). An online survival analysis tool to rapidly assess the effect of 22,277 genes on breast cancer prognosis using microarray data of 1,809 patients. *Breast Cancer Res. Treat.* 123 725–731. 10.1007/s10549-009-0674-9 20020197

[B26] Herman J. G. Baylin S. B. (2003). Gene silencing in cancer in association with promoter hypermethylation. *N. Engl. J. Med.* 349 2042–2054. 10.1056/nejmra023075 14627790

[B27] Huang C. C. Tu S. H. Lien H. H. Jeng J. Y. Huang C. S. Huang C. J. (2013). Concurrent gene signatures for han chinese breast cancers. *PLoS One* 8:e76421. 10.1371/journal.pone.0076421 24098497 PMC3789693

[B28] Huang Y. S. Liu W. B. Han F. Yang J. T. Hao X. L. Chen H. Q. (2017). Copy number variations and expression of MPDZ are prognostic biomarkers for clear cell renal cell carcinoma. *Oncotarget* 8 78713–78725. 10.18632/oncotarget.20220 29108259 PMC5667992

[B29] Iglesia M. D. Parker J. S. Hoadley K. A. Serody J. S. Perou C. M. Vincent B. G. (2016). Genomic analysis of immune cell infiltrates across 11 tumor types. *J. Natl. Cancer Inst.* 108:djw144. 10.1093/jnci/djw144 27335052 PMC5241901

[B30] Janeway C. J. Travers P. Walport M. Shlomchik M. J. (2001). *Immunobiology: The Immune System in Health and Disease*, 9th Edn New York, NY: Garland Science, 924.

[B31] Jin H. Huang X. Shao K. Li G. Wang J. Yang H. (2019). Integrated bioinformatics analysis to identify 15 hub genes in breast cancer. *Oncol. Lett.* 18 1023–1034. 10.3892/ol.2019.10411 31423162 PMC6607081

[B32] Karsli-Ceppioglu S. Dagdemir A. Judes G. Lebert A. Penault-Llorca F. Bignon Y. J. (2017). The epigenetic landscape of promoter genome-wide analysis in breast cancer. *Sci. Rep.* 7:6597. 10.1038/s41598-017-06790-z 28747748 PMC5529370

[B33] Kim I. Choi S. Kim S. (2018). BRCA-pathway: a structural integration and visualization system of TCGA breast cancer data on KEGG pathways. *BMC Bioinformatics* 19(Suppl. 1):42. 10.1186/s12859-018-2016-6 29504910 PMC5836821

[B34] Lance G. N. Williams W. T. (1967). A general theory of classificatory sorting strategies: 1. Hierarchical Systems. *Comput. J.* 9 373–380. 10.1093/comjnl/9.4.373

[B35] Li B. Geng R. Wu Q. Yang Q. Sun S. Zhu S. (2020). Alterations in immune-related genes as potential marker of prognosis in breast cancer. *Front. Oncol.* 10:333. 10.3389/fonc.2020.00333 32226776 PMC7080956

[B36] Luen S. Virassamy B. Savas P. Salgado R. Loi S. (2016). The genomic landscape of breast cancer and its interaction with host immunity. *Breast* 29 241–250. 10.1016/j.breast.2016.07.015 27481651

[B37] Mathews J. C. Nadeem S. Levine A. J. Pouryahya M. Deasy J. O. Tannenbaum A. (2019). Robust and interpretable PAM50 reclassification exhibits survival advantage for myoepithelial and immune phenotypes. *NPJ Breast Cancer* 5:30. 10.1038/s41523-019-0124-8 31531391 PMC6733897

[B38] Netanely D. Avraham A. Ben-Baruch A. Evron E. Shamir R. (2016). Expression and methylation patterns partition luminal-A breast tumors into distinct prognostic subgroups. *Breast Cancer Res.* 18:74. 10.1186/s13058-016-0724-2 27386846 PMC4936004

[B39] Nguyen H. Shrestha S. Draghici S. Nguyen T. (2018). PINSPlus: a tool for tumor subtype discovery in integrated genomic data. *Bioinformatics* 35 2843–2846. 10.1093/bioinformatics/bty1049 30590381

[B40] Nguyen T. Tagett R. Diaz D. Draghici S. (2017). A novel approach for data integration and disease subtyping. *Genome Res.* 27 2025–2039. 10.1101/gr.215129.116 29066617 PMC5741060

[B41] Ogata H. Goto S. Sato K. Fujibuchi W. Bono H. Kanehisa M. (1999). KEGG: kyoto encyclopedia of genes and genomes. *Nucleic Acids Res.* 27 29–34. 9847135 10.1093/nar/27.1.29 PMC148090

[B42] Parker J. S. Mullins M. Cheang M. C. Leung S. Voduc D. Vickery T. (2009). Supervised risk predictor of breast cancer based on intrinsic subtypes. *J. Clin. Oncol.* 27 1160–1167. 10.1200/jco.2008.18.1370 19204204 PMC2667820

[B43] Perou C. M. Sørlie T. Eisen M. B. van de Rijn M. Jeffrey S. S. Rees C. A. (2000). Molecular portraits of human breast tumours. *Nature* 406 747–752. 10963602 10.1038/35021093

[B44] Rappoport N. Shamir R. (2018). Multi-omic and multi-view clustering algorithms: review and cancer benchmark. *Nucleic Acids Res.* 46 10546–10562. 10.1093/nar/gky889 30295871 PMC6237755

[B45] Rice J. C. Ozcelik H. Maxeiner P. Andrulis I. Futscher B. W. (2000). Methylation of the BRCA1 promoter is associated with decreased BRCA1 mRNA levels in clinical breast cancer specimens. *Carcinogenesis* 21 1761–1765. 10.1093/carcin/21.9.1761 10964110

[B46] Richard F. Gengelbach M. P. Schlüns K. Fleige B. Winzer K. J. Szymas J. (2000). Patterns of chromosomal imbalances in invasive breast cancer. *Int. J. Cancer* 89 305–310. 10.1002/1097-0215(20000520)89:3<305::aid-ijc15>3.0.co;2-8 10861509

[B47] Rønneberg J. A. Fleischer T. Solvang H. K. Nordgard S. H. Edvardsen H. Potapenko I. (2011). Methylation profiling with a panel of cancer related genes: association with estrogen receptor, TP53 mutation status and expression subtypes in sporadic breast cancer. *Mol. Oncol.* 5 61–76. 10.1016/j.molonc.2010.11.004 21212030 PMC5528272

[B48] Russnes H. G. Vollan H. K. M. Lingjærde O. C. Krasnitz A. Lundin P. Naume B. (2010). Genomic architecture characterizes tumor progression paths and fate in breast cancer patients. *Sci. Transl. Med.* 2:38ra47. 10.1126/scitranslmed.3000611 20592421 PMC3972440

[B49] Samulin Erdem J. Arnoldussen Y. J. Skaug V. Haugen A. Zienolddiny S. (2017). Copy number variation, increased gene expression, and molecular mechanisms of neurofascin in lung cancer. *Mol. Carcinog.* 56 2076–2085. 10.1002/mc.22664 28418179 PMC6084301

[B50] Shen H. Laird P. W. (2013). Interplay between the cancer genome and epigenome. *Cell* 153 38–55. 10.1016/j.cell.2013.03.008 23540689 PMC3648790

[B51] Shen R. Mo Q. Schultz N. Seshan V. E. Olshen A. B. Huse J. (2012). Integrative subtype discovery in glioblastoma using iCluster. *PLoS One* 7:e35236. 10.1371/journal.pone.0035236 22539962 PMC3335101

[B52] Shen R. Olshen A. B. Ladanyi M. (2009). Integrative clustering of multiple genomic data types using a joint latent variable model with application to breast and lung cancer subtype analysis. *Bioinformatics* 25 2906–2912. 10.1093/bioinformatics/btp543 19759197 PMC2800366

[B53] Shi X. Zhao Q. Huang J. Xie Y. Ma S. (2015). Deciphering the associations between gene expression and copy number alteration using a sparse double Laplacian shrinkage approach. *Bioinformatics* 31 3977–3983. 10.1093/bioinformatics/btv518 26342102 PMC5013934

[B54] Stephens P. J. Tarpey P. S. Davies H. Van Loo P. Greenman C. Wedge D. C. (2012). The landscape of cancer genes and mutational processes in breast cancer. *Nature* 486 400–404. 10.1038/nature11017 22722201 PMC3428862

[B55] Timothy C. U. (2017). *Statistics in Plain English*, 4 Edn Abingdon: Routledge.

[B56] Untch M. Harbeck N. Huober J. von Minckwitz G. Gerber B. Kreipe H. H. (2015). Primary therapy of patients with early breast cancer: evidence, controversies, consensus: opinions of german specialists to the 14th St. gallen international breast cancer conference 2015 (Vienna 2015). *Geburtshilfe Frauenheilkd.* 75 556–565. 26166836 10.1055/s-0035-1546120 PMC4490924

[B57] Xia Q. Li Z. Zheng J. Zhang X. Di Y. Ding J. (2019). Identification of novel biomarkers for hepatocellular carcinoma using transcriptome analysis. *J. Cell. Physiol.* 234 4851–4863. 10.1002/jcp.27283 30272824

[B58] Xia Y. Fan C. Hoadley K. A. Parker J. S. Perou C. M. (2019). Genetic determinants of the molecular portraits of epithelial cancers. *Nat. Commun.* 10:5666. 10.1038/s41467-019-13588-2 31827079 PMC6906458

[B59] Xu M. Li Y. Li W. Zhao Q. Zhang Q. Le K. (2020). Immune and stroma related genes in breast cancer: a comprehensive analysis of tumor microenvironment based on the cancer genome atlas (TCGA) database. *Front. Med.* 7:64. 10.3389/fmed.2020.00064 32195260 PMC7066229

[B60] Xu T. Le T. D. Liu L. Su N. Wang R. Sun B. (2017). CancerSubtypes: an R/Bioconductor package for molecular cancer subtype identification, validation and visualization. *Bioinformatics* 33 3131–3133. 10.1093/bioinformatics/btx378 28605519

[B61] Yang Z. Liu B. Lin T. Zhang Y. Zhang L. Wang M. (2019). Multiomics analysis on DNA methylation and the expression of both messenger RNA and microRNA in lung adenocarcinoma. *J. Cell. Physiol.* 234 7579–7586. 10.1002/jcp.27520 30370535

[B62] Zhou C. Zhang W. Chen W. Yin Y. Atyah M. Liu S. (2017). Integrated analysis of copy number variations and gene expression profiling in hepatocellular carcinoma. *Sci. Rep.* 7:10570. 10.1038/s41598-017-11029-y 28874807 PMC5585301

