# Supplementary material for: Multi-Omics Analysis Detects Novel Prognostic Subgroups of Breast Cancer
Source: Front Genet. 2020 Oct 15;11:574661. doi: 10.3389/fgene.2020.574661 (PMC7594512; doi:10.3389/fgene.2020.574661)
Supplement: Supplementary file 2 [file Data_Sheet_2.pdf]

Supplementary Figures

A

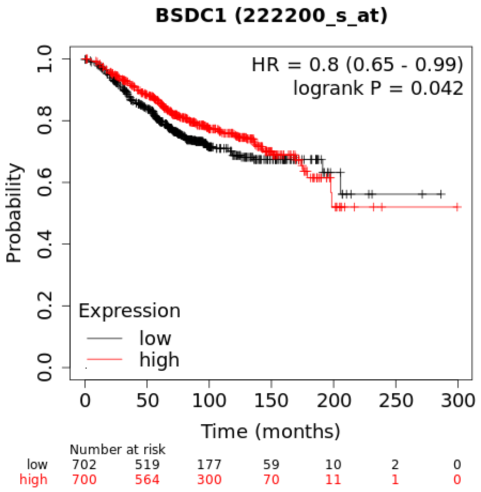

B

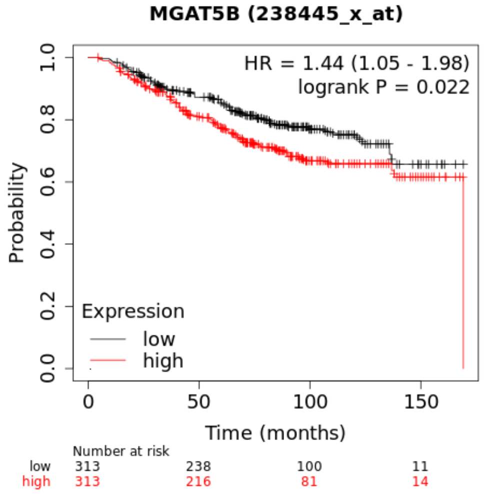

C

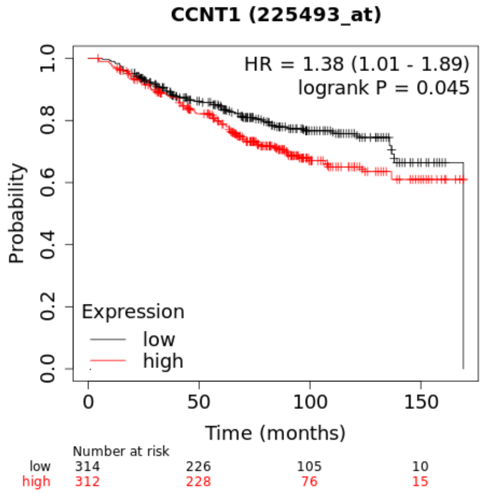

D

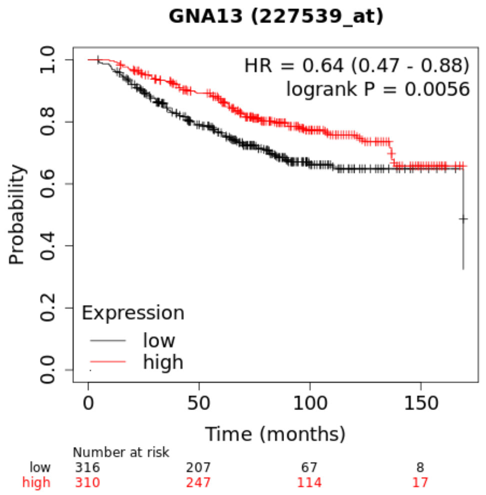
GNA13 (227539_at)
HR = 0.64 (0.47 - 0.88)
logrank P = 0.0056
Probability
0.0 0.2 0.4 0.6 0.8 1.0
Expression
low
high
0 50 100 150
Time (months)
Number at risk
low 316 207 67 8
high 310 247 114 17

E

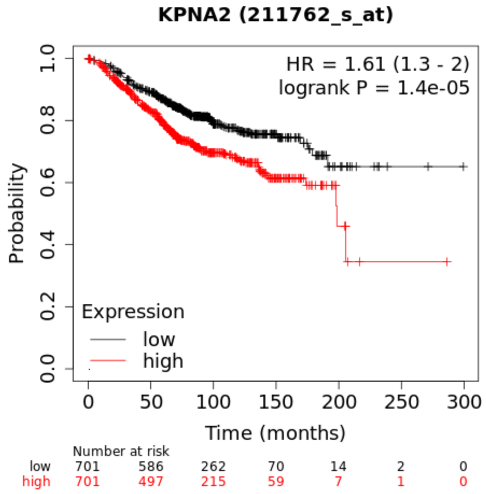
KPNA2 (211762_s_at)
HR = 1.61 (1.3 - 2)
logrank P = 1.4e-05
Probability
0.0 0.2 0.4 0.6 0.8 1.0
Expression
low
high
0 50 100 150 200 250 300
Time (months)
Number at risk
low 701 586 262 70 14 2 0
high 701 497 215 59 7 1 0

F

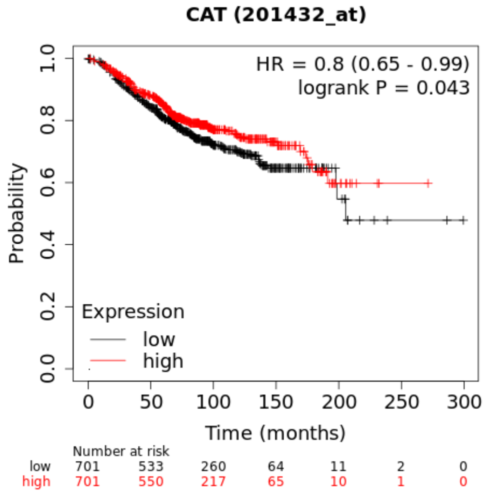
CAT (201432_at)
HR = 0.8 (0.65 - 0.99)
logrank P = 0.043
Probability
0.0 0.2 0.4 0.6 0.8 1.0
Expression
low
high
0 50 100 150 200 250 300
Time (months)
Number at risk
low 701 533 260 64 11 2 0
high 701 550 217 65 10 1 0

G

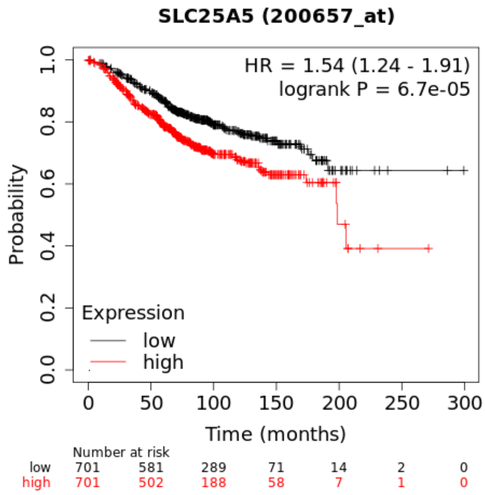

Figure S1. Kaplan-Mayer survival curves in the KMplot dataset. (A-E) Biomarker genes in the CNAcor gene sets (F-G) Biomarker genes in the METcor gene sets.

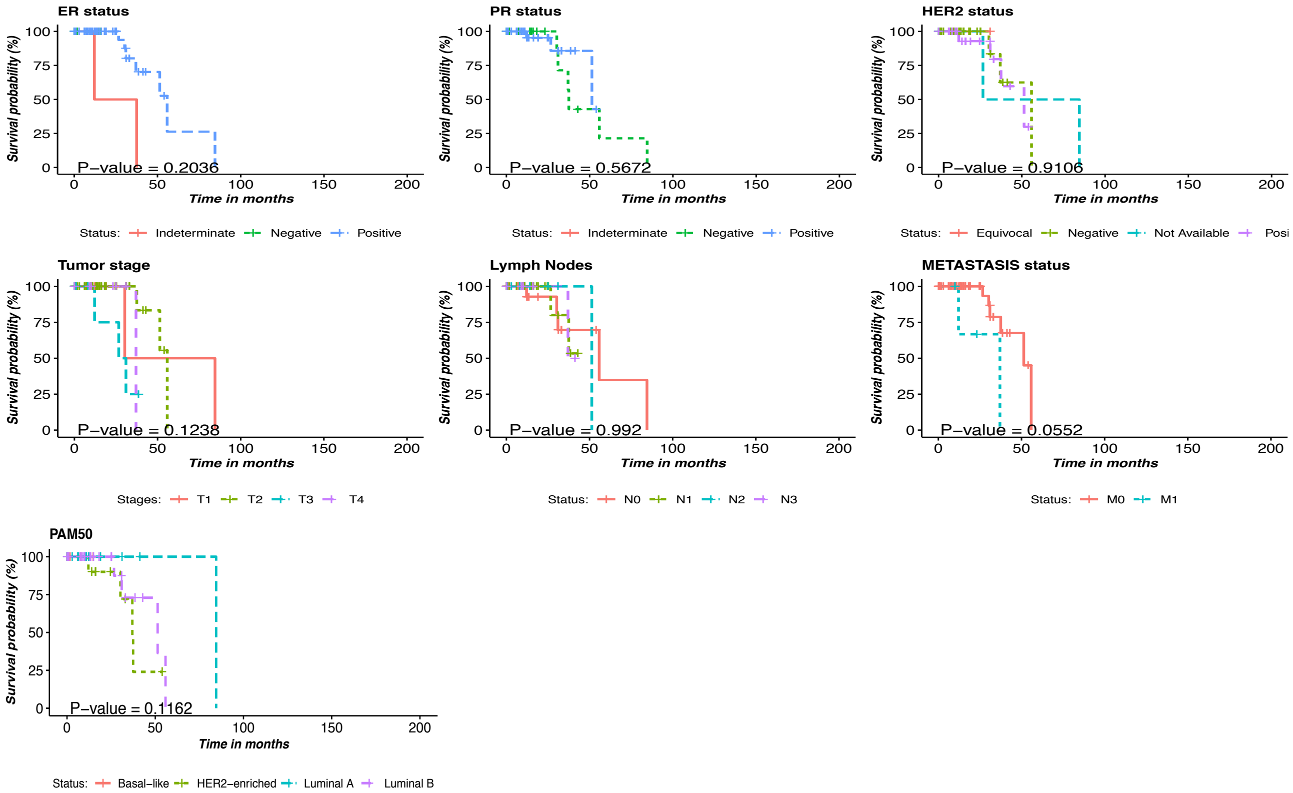

Figure S2. IntCl2-clinical features correlations. Correlation of the IntCl2 with other clinical features, including ER status, PR status, HER2 status, Tumor stage, Lymph nodes, METASTASIS, and PAM50 scheme. All of them are not statistically significant (i.e., P-value > 0.05).

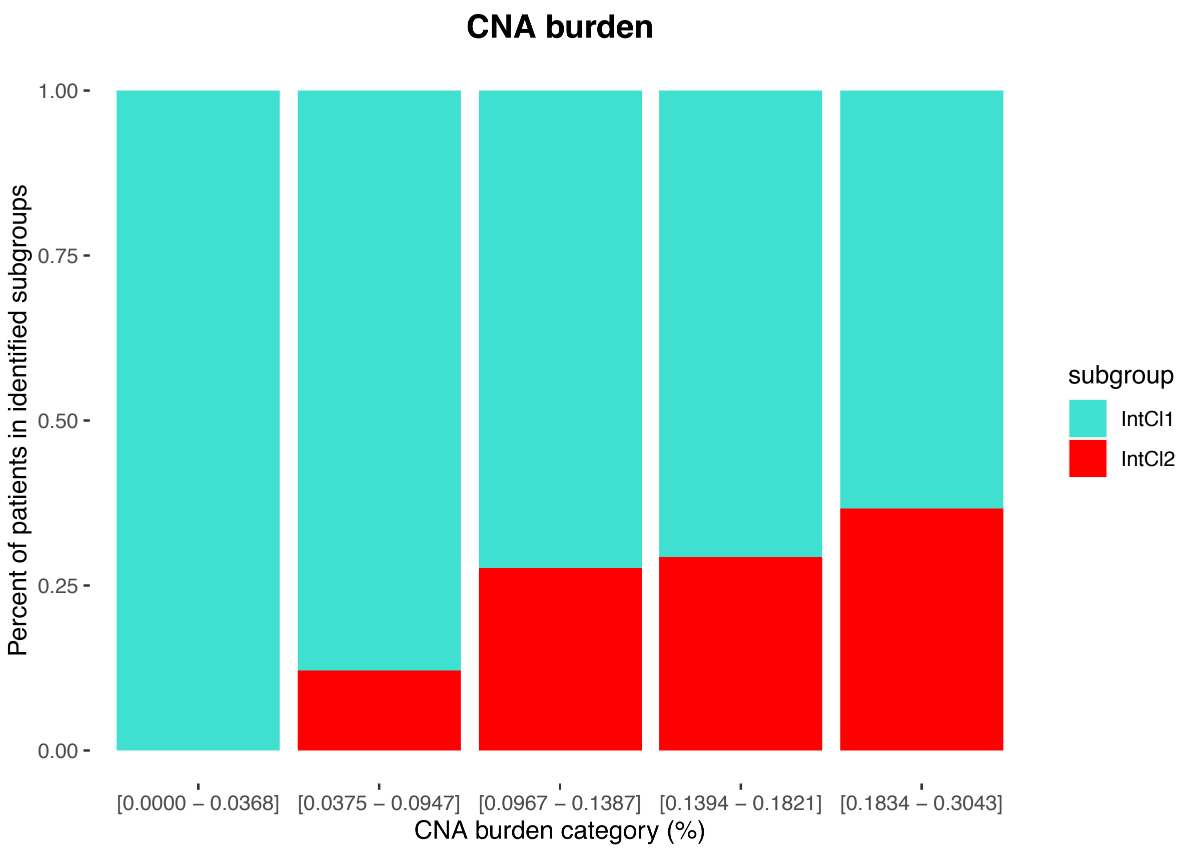

Figure S3. CNA burden by quintiles within the two subgroups. CNA burden by the two identified subgroups in the discovery data.
